# Exploring the attitudes and practices among student clinicians in India on multilingual issues in the field of speech-language pathology

**DOI:** 10.1590/2317-1782/20232022249en

**Published:** 2023-09-01

**Authors:** Indira Valliappan, Roha Kaipa, Sudhin Karuppali

**Affiliations:** 1 Department of Audiology and Speech Language Pathology, Kasturba Medical College, Mangalore, Manipal; Academy of Higher Education, Manipal, India.; 2 Department of Communication Sciences and Disorders, Social Sciences and Humanities, Oklahoma State University - Stillwater (Oklahoma), United States.

**Keywords:** Clinician, Issues, Language, Multilingual, Student

## Abstract

**Purpose:**

Student clinicians (graduates and undergraduates) in speech-language pathology deal with various multilingual issues while providing clinical services to individuals with language impairments. This study explores the attitudes and practices of undergraduate and graduate speech-language pathology students in India towards multilingualism and handling these issues.

**Methods:**

One hundred and twenty-eight students (71 graduates and 57 undergraduates) participated in the study. Phase 1 of the study included the development of a questionnaire to explore the attitudes and practices of student clinicians in speech-language pathology. The questionnaire was converted into an online survey in Phase 2. Phase 3 comprised data and statistical analysis to summarize and interpret collected data.

**Results:**

Graduate and undergraduate students significantly differed in their attitudes and perception toward multilingual issues (p<0.05). Most clinicians demanded a change in the current views on assessment/intervention, considering the linguistic background of the patient/caregivers. Other issues surrounding multilingualism included parents' education levels, lack of sufficient assessment tools, unavailability of translators/interpreters, and poor linguistic competency of clinicians.

**Conclusion:**

These findings assist academic programs in planning and developing modules to aid students in handling the major multilingual issues encountered during clinical interactions.

## INTRODUCTION

Multilingualism is a complex phenomenon and has been an area of interest across various disciplines, including linguistics, education, speech-language pathology, and others^(1)^. A multilingual uses more than one language for communication, either actively (speaking and writing) or passively (listening and reading). Instructional models, such as the general curricular infusion or dedicated instructional model, often address the multilingual issues encountered by graduate and undergraduate students in speech-language pathology. The term student-clinician is a generic term used for either undergraduate or graduate students in speech-language pathology providing clinical services to individuals with language impairments under the direct supervision of experienced speech-language pathologists (SLPs). The general curricular infusion model includes adding or replacing multilingual issues to the existing course content. In contrast, the dedicated instructional model incorporates courses that address multilingual issues. The American Speech–Language and Hearing Association (ASHA) enforces certain foundational courses to address the multilingual and multicultural issues that students may encounter. Accent modification, aphasia, adult neurogenic communication disorders, fluency and voice disorders, dysphagia, and stuttering are a few academic courses that cover multilingual and multicultural issues. Such programs enable the students to deal with situations involving multilingual patients better^(2)^. The role of the SLP in differentiating language impairments from language differences is fundamental. While carrying out language assessment in a multilingual client, the SLP needs to determine if the language deficits manifest in all their languages. Language experiences and environmental factors are critical in assessment and intervention planning. Bilingual patients from bilingual environments prefer to receive bilingual intervention and require adequate tools for assessment/intervention of language impairments, access to interpretive/translating services, and adequately trained personnel.

Williams and McLeod^(3)^ explored the knowledge and practices of Australian SLPs in assessing and intervening children who speak a language other than English. Half of the 128 SLPs who participated reported minimal proficiency in languages other than English. Roughly 10% of the participants reported being competent in another language. The SLPs reported that in the past 12 months, they had an average of 59.2 (range of 1-100) clients on the caseload who spoke a different language than English. Most of these clients required speech (50.5%) and language assessments (34.2%), and the SLPs carried them out without assistance from interpreters. Informal assessments were carried out predominantly (speech-76.7 and language-78.2%), and English standardized assessments were administered when needed. The participants reported needing additional information about the client's language and cultural backgrounds to distinguish differences versus disorders, but they were limited on resources available^(3)^. Another study by Verdon et al.^(4)^ focused on the geographical analysis of speech-language services provided to multilingual children in Australia. The authors reported that the proportion of Australian SLPs offering services in languages (Italian and French) other than English had significantly increased over time. Although SLPs provided services in languages other than English, there was a discrepancy between the place of residence of the multilingual children and the languages in which multilingual services were offered. The authors recommended pre-service and in-service training to prepare monolingual and multilingual SLPs to practice efficiently in multilingual contexts. The authors also stated the considerations for choosing a particular language for intervention were the language spoken by the child, the language spoken by SLPs, the language spoken at school, the language spoken in the community, and the language insisted by the parent.

Glazzard^(5)^ was against a direct translation of standardized assessment tools and insisted on the translation and development of assessment tools that may meet the local norms of the region. He promoted dynamic assessment, i.e., differentiating children with language impairments from typically developing multilingual children. Accordingly, SLPs developed an understanding of cultural assumptions imbibed in them, attempting to increase their knowledge of perceptions of other cultural groups. Bove^(6)^ found that graduate students' perceptions of working with people from diverse cultural and linguistic backgrounds changed after joining a multicultural-based course. Students developed a feeling of clinical competency, with their understanding and confidence (from neutral to strongly agree) in dealing with diverse populations gradually growing by linking real-life experiences with their practice. In a recent study, Getzler et al.^(7)^ found monolingual SLPs to be largely unprepared to work and communicate with bilingual children and felt that the coursework available for SLPs to handle bilingual children was limited, indicating a change in the curriculum and treatment methods to adapt to this population. A systematic review^(8)^ reported that monolingual SLPs feel inadequately prepared to support patients who use a language different than their own compared to bilingual SLPs. This included a poor understanding of the linguistic and cognitive developmental norms, the unavailability of appropriate linguistic assessment tools, and the lack of skilled language interpreters. An increase in the access and availability of pre-service (practicum and the coursework that can be completed in graduate school) and in-service (ongoing on-the-job continued education, workshops, and professional collaborations) programs for practicing professionals were noted.

India has a rich cultural and linguistic diversity of 447 documented languages and several regional dialectical variations. This cultural and linguistic diversity has shaped traditional customs, social norms, beliefs, political systems, and ethical values. The Indian languages belong to the Indo-European and Dravidian language families. The languages spoken across the country largely depend on the geographical location of the speaker. Migrants from one part of the country learn the regional languages to fully immerse themselves in the new community and eventually become multilinguals. Nevertheless, multilingualism need not be attained only through the migratory process but can also be achieved through individuals residing in geographical regions with multiple local languages. With multilingualism being a norm in India, it is majorly attributed to the individual's geographical rather than the socioeconomic background, with the former having an immense influence on language proficiency levels^(9)^. However, there are no established Indian studies on the influence of socioeconomic background over the mastery of languages, as may be observed in western countries.

SLPs in India, compared to western countries, handle patients from diverse linguistic, educational, and economic backgrounds. However, the level of preparedness during such clinical encounters is less due to the lack of multilingual and multicultural instructional courses infused into the speech-language pathology curriculum in India. With the lack of such curricular programs in India, it becomes vital to explore the challenges and barriers Indian SLPs face while encountering multilingual patients during assessment and intervention. Many clinics in India adopt assessments and tools standardized in western countries, resulting in overestimating the actual extent of the disability, largely due to the linguistic variability between English and the Indian languages (scripted/unscripted/tonal/non-tonal). Undergraduate student-clinicians of speech-language pathology in India start observing clinical sessions right from the first semester of their bachelor's program. By the second semester, they join the senior student and become active co-clinicians. These students tend to be independent clinicians from their second year and complete a 10-month externship in the bachelor's program during the fourth year. On the other hand, the graduate student-clinicians of speech-language pathology assess and intervene clients under supervision from their first semester. As per the curriculum determined by the Rehabilitation Council of India (RCI), undergraduate student clinician needs a total of 1800 clinical hours during their first six semesters and 1260 clinical hours during their externship. Similarly, a graduate student clinician needs 800 clinical hours during their two-year graduate program. The students obtain these clinical hours under faculty supervision and are mandatory to meet the regulatory guidelines set by RCI^(10)^.

The current study explored the multilingual issues faced by speech-language pathology students in India handling diverse languages and dialectal variations. A culturally and linguistically sensitive questionnaire/survey was developed to explore the multilingual issues faced by undergraduate and graduate students in speech-language pathology. The survey was distributed among undergraduate and graduate students between March 2021 and February 2022.

## METHOD

The current study aimed to explore the attitudes and practices of speech-language pathology students in India on multilingual issues. The Institutional Ethics Committee approved the study protocol (IEC KMC MLR 03-2021/100) of Kasturba Medical College, Mangalore, Manipal Academy of Higher Education. A non-random convenience sampling was adopted for the study.

### Participants

The sample size was calculated using n=Z^2^_1-α/2_ p(1-p)/(d)^2^, where $Z_{1-\alpha /2}^{}\;$ **=** 1.96, p= 0.5 and d= 0.05. The sample size was estimated from a previous survey of similar nature^(2)^. Of the 180 students contacted, 128 participants responded and were included in the current study. The demographic details of the participant are provided in Table 1.

**Table 1:** The demographic details of the participants

Educational level	Gender		Total	Mean age
	Males	Females		
	N (%)	N (%)	N (%)	(in years)
UG	4 (5.6)	67 (94.40)	71	21.41(±1.06)
PG [M.Sc (SLP)]	3 (6.8)	41 (93.2)	44	23.5 (±0.86)
PG (MASLP)	1(7.69)	12 (92)	13	23.8 (±1.3)
Total	8 (6.2)	120 (93.8)	128	22.64 (±1.47)

**Caption:** UG – Final year undergraduates; PG – Final year postgraduates; M.Sc (SLP) – Masters in Science (Speech-Language Pathology); MASLP – Masters in Audiology and Speech-Language Pathology

The final-year undergraduates and final-year graduates enrolled in speech-language pathology participated in the study. All participants were recruited from various speech and hearing colleges/universities across India. The participants were pursuing their education in ten different cities in India, including Mangalore (33%), Mysore (30%), Chennai (16%), Bangalore, Manipal, Trivandrum (5%), Kannur, Pune, Thrissur, and Surat (2% and less).

The current study recruited final-year undergraduate and final-year graduate students pursuing speech-language pathology in any RCI-recognized academic institution in India. Students with clinical experience other than the experiences they received from their graduate program and students with an additional educational degree were excluded from the study. All selected participants signed the consent form before they participated in the study. The table 2 below illustrates the language background of the participants collected from questions 3-7 of the survey.

**Table 2:** Language proficiency ratings of the participants based on the number of languages known.

Number of languages known	Levels of proficiency	Languages	The total proportion of participants		
			Reading n (%)	Writing n (%)	Speaking n (%)
1	0-2	L1	2 (1.5)	2 (1.5)	-
	3-5	L1	-	1 (0.8)	-
	6-8	L1	3 (2.3)	4 (3.1)	-
	9-10	L1	5 (3.9)	5 (3.9)	5 (3.9)
2	0-2	L1	-	-	-
		L2	1 (0.8)	1 (0.8)	-
	3-5	L1	-	-	1 (0.8)
		L2	1 (0.8)	1 (0.8)	2 (1.5)
	6-8	L1	9 (7)	10 (7.8)	11 (8.6)
		L2	9 (7)	13 (10.2)	7 (5.4)
	9-10	L1	25 (19.5)	27 (21)	12 (9.3)
		L2	23 (18)	21 (16.4)	15 (11.7)
3	0-2	L1	-	1 (0.8)	-
		L2	2 (1.5)	2 (1.5)	1 (0.8)
		L3	2 (1.5)	3 (2.3)	1 (0.8)
	3-5	L1	1 (0.8)	-	1 (0.8)
		L2	1 (0.8)	5	2 (1.5)
		L3	7 (5.4)	9 (7)	4 (3.1)
	6-8	L1	1 (0.8)	3 (2.3)	6 (4.7)
		L2	15 (11.7)	16 (12.5)	6 (4.7)
		L3	27 (21)	25 (19.5)	13 (10.2)
	9-10	L1	43 (33.6)	43 (33.6)	18 (14)
		L2	29 (22.7)	25 (19.5)	16 (12.5)
		L3	11 (8.6)	10 (7.8)	7 (5.4)
4	0-2	L1	-	-	-
		L2	5 (3.9)	4 (3.1)	-
		L3	3 (2.3)	2 (1.5)	-
		L4	1 (0.8)	2 (1.5)	1 (0.8)
	3-5	L1	-	1 (0.8)	-
		L2	1 (0.8)	-	8 (6.25)
		L3	1 (0.8)	3 (2.3)	13 (10.2)
		L4	5 (3.9)	7 (5.4)	9 (7)
	6-8	L1	5 (3.9)	3 (2.3)	16 (12.5)
		L2	6 (4.7)	6 (4.7)	13 (10.2)
		L3	10 (7.8)	7 (5.4)	15 (11.7)
		L4	14 (10.9)	8 (6.25)	21 (16.4)
	9-10	L1	25 (19.5)	27 (21)	27 (21)
		L2	15 (11.7)	11 (8.6)	18 (14)
		L3	13 (10.2)	11 (8.6)	19 (14.8)
		L4	8 (6.25)	9 (7)	9 (7)
>4	0-2	L1	-	1 (0.8)	-
		L2	2 (1.5)	-	-
		L3	1 (0.8)	1 (0.8)	3 (2.3)
		L4	2 (1.5)	4 (3.1)	1 (0.8)
		L5	1 (0.8)	1 (0.8)	6 (4.7)
		L6	1 (0.8)	1 (0.8)	2 (1.5)
		L7	-	-	-
		L8	-	-	1 (0.8)
	3-5	L1	-	-	-
		L2	-	1 (0.8)	6 (4.7)
		L3	2 (1.5)	-	4 (3.1)
		L4	2 (1.5)	-	4 (3.1)
		L5	-	1 (0.8)	7 (5.4)
		L6	-	1 (0.8)	9 (7)
		L7	-	-	2 (1.5)
		L8	-	-	1 (0.8)
	6-8	L1	1 (0.8)	-	8 (6.25)
		L2	2 (1.5)	-	13 (10.2)
		L3	1 (0.8)	2 (1.5)	19 (14.8)
		L4	1 (0.8)	1 (0.8)	15 (11.7)
		L5	9 (7)	-	15 (11.7)
		L6	-	-	-
		L7	-	-	1 (0.8)
		L8	-	-	-
	9-10	L1	6 (4.7)	5 (3.9)	27 (21)
		L2	2 (1.5)	3 (2.3)	14 (11)
		L3	3 (2.3)	2 (1.5)	7 (5.4)
		L4	5 (3.9)	2 (1.5)	13 (10.2)
		L5	2 (1.5)	1 (0.8)	6 (4.7)
		L6	-	-	3 (2.3)
		L7	-	-	1 (0.8)
		L8	-	-	-

**Caption:** Languages-L1: First language, L2: Second language, L3: Third language, L4: Fourth language, L5: Fifth language, L6: Sixth language, L7: Seventh language, L8: Eighth language. Levels of proficiency-0: none, 1: very low, 2: low, 3: fair, 4: slightly less than adequate, 5: adequate, 6: slightly more than adequate, 7: good, 8: very good, 9: excellent, 10: perfect

### Procedure

The current study was conducted in three phases. Phase I was the questionnaire development, Phase 2 was data collection, and Phase 3 comprised data and statistical analysis.

### Phase I: Development of the questionnaire

The questionnaire collected the demographics, the nature of multilingual issues encountered by student clinicians during the assessment and intervention of patients, and strategies used by students to handle such multilingual issues. The authors sought permission from Stockman et al.^(2)^ to adopt some questions from their study. The questionnaire authors developed included 34 items, out of which ten were adapted from Stockman et al.^(2).^ The rest 24 were developed after reviewing the literature on multilingualism. All 34 items were subjected to content validation by three experienced SLPs with a minimum of 5 years of clinical experience using a 4-point rating scale (0-very inappropriate, 1-inappropriate, 2-appropriate, and 3-very appropriate). The questionnaire was modified and consolidated based on the feedback. The final questionnaire comprised 23 items, including 19 multiple-choice items, two Likert scale ratings, and two open-ended items. The final questionnaire obtained a content validation index of 0.91. The final questionnaire is attached in the Appendix 1.

### Phase 2: Data collection

The questionnaire was converted into an online survey using Qualtrics^XM^. The survey link was distributed along with a brief study description to final-year undergraduate and graduate student clinicians. As soon as the participants clicked the survey link, they landed on the consent form and study description. Once the participant provided consent, they could proceed to the next page and complete the survey. Each participant took around 15 minutes to complete the survey. Two reminders were sent via email to encourage participants to complete the survey: one on the seventh day and the second on the ninth day.

### Phase 3: Data and statistical analysis

The collected data was downloaded from Qualtrics^XM^ for data analysis. Descriptive statistics were used for analyzing the data using SPSS (version 16.0). Chi-square tests were carried out to determine the strength of association between the parameters of interest.

## RESULTS

One hundred twenty-eight participants responded to the survey, yielding a response rate of 71.11%. The responses of the participants are discussed below.

### Clinicians' perception of multilingual assessment and intervention

**Figure 1:**
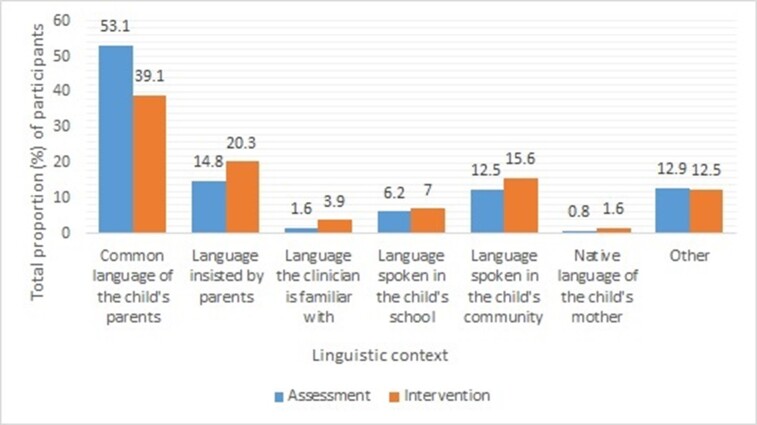
The language used by the student clinicians for the assessment/intervention of multilingual children based on the linguistic context

Questions 10-13 of the survey collected clinicians' perceptions of multilingual assessment and intervention. Likert scale ratings were employed to collect this information. Around 101 (79%) participants felt the need to select assessment and treatment depending on the linguistic background of the client and rated 'always' and 'most of the time' on the Likert scale. However, 25 (19.5%) and 19 (14.8%) participants selected 'sometimes' to the above question regarding assessment and treatment, respectively. Figure 1 illustrates the proportion of student clinicians who preferred to use a particular language for the assessment/intervention while serving multilingual clients.

### Factors/Issues surrounding the assessment and intervention of multilingual children with language impairment

Factors/issues surrounding the assessment and intervention of multilingual children with language impairment were collected using questions 8, 9, 14 to 21. A total of 84 (65.6%) student clinicians reported that the education of parents did affect both assessment and intervention; 26 (20.3%) reported that it neither affected assessment nor intervention; 15 (11.7%) reported that it affected only intervention, while 3 (2.3%) reported it affected assessment. Additionally, 74 (57.8%) participants reported that the available multilingual tools were not sufficient for assessment, 9 (7%) reported otherwise, and 45 (35.2%) were uncertain about the same. Table 3 represents the communication breakdowns the participants experienced while handling multilingual children with language impairments.

**Table 3:** Perceptions of student clinicians towards encountering communication breakdowns with multilingual children with language impairment

Items	Total proportion [n (%)] of student clinicians			
	0-24%	25-49%	50-74%	75-100%
How often do you face a communication breakdown due to multilingual challenges during the assessment of children with language impairment? (Q14)	37 (28.9)	47 (36.7)	38 (29.7)	6 (4.7)
How often do you face a communication breakdown due to multilingual challenges during the intervention of children with language impairment? (Q15)	37 (28.9)	49 (38.3)	34 (26.6)	8 (6.2)
How often did the child misunderstand your instructions during a communication breakdown? (Q16)	66 (51.6)	43 (33.6)	17 (13.3)	2 (1.6)
How often did the child’s parent misunderstand your instructions? (Q17)	61 (47.7)	44 (34.4)	22 (17.8)	1 (0.8)
How often did you experience a communication breakdown even though a translator was available? (Q20)	71 (64.5)	25 (22.7)	13 (11.8)	1 (1)

Although 18 (14.1%) participants reported managing with translation services, 82 (64%) reported the need for a translator for assessment and intervention, while 18 (14.1%) and 10 (7.8%) reported the need for a translator only for assessment and intervention services, respectively. Sixty-three (57.3%) participants reported having a co-clinician as the translator, 43 (39.1%) reported having a family member as the translator, and 4 (3.6%) opted for other means. Additionally, 48 (43.6%) participants mentioned encountering situations where the child and the clinician did not share a common language while a translator was unavailable.

### Preparedness of graduate and undergraduate student clinicians in assessing and treating multilingual children with language impairment

Questions 7, 22, and 23 collected information on the preparedness of student clinicians in assessing and treating multilingual clients. Fifty-eight (45.3%) reported that their patients spoke a language in which the clinician was proficient 75-100% of the time, 42 (32.8%) felt 50-74% of the time, 20 (15.6%) felt 25-49% of the times, and 8 (6.2%) felt 0-24% of the times. A large proportion of participants, 78 (60.9%), felt ‘well prepared’ to deal with the multilingual issues they encountered in the clinic, compared to 48 (39%) who were 'unsure' or felt poorly prepared to handle these issues. Eight-two (64%) participants described that providing clinical services to multilingual population did eventually help them to handle multilingual issues efficiently. In comparison, 57 (44.5%) mentioned that following a mentor/colleague's recommendations helped them, 49 (38.3%) reported completing continuing education programs, and 36 (28%) participants asserted that researching such topics benefited them in handling multilingual issues.

### Perceptions of multilingual issues among undergraduate and graduate students

The authors also compared the perceptions of undergraduate and graduate clinicians toward multilingualism. Table 4 illustrates the total number of undergraduates and graduates and their chi-square correlation across parameters of concern.

**Table 4:** The total number of undergraduates and graduates and their chi-square correlation across parameters of concern

Sl. No	Items	Undergraduates n(%)	Graduates n(%)	Value
1.	Existence of variation in assessment based on whether the child is mono/bi/ multilingual (Always)	17 (23.9)	31(54.4)	ꭓ^2^(4)= 16.027=0.003, p<0.05
2.	Existence of variation in intervention based on whether the child is mono/bi/ multilingual (Always)	23(32.4)	33(57.9)	ꭓ^2^(4)= 9.993=0.041, p<0.05
3.	Importance of learning about multilingual issues (Very important)	31(43.7)	38(66.7)	ꭓ^2^(3)= 8.703=0.034, p<0.05
4.	Level of preparation to apply the knowledge about multilingual issues (Exceptionally well prepared)	4(5.6)	14(24.6)	ꭓ^2^(4)= 11.955=0.018, p<0.05

## DISCUSSION

The current study explored the attitudes and practices of speech-language pathology students in India on multilingual issues encountered during their clinical interactions. The discussion follows the same outline as that of the results.

### Clinicians' perception of multilingual assessment and intervention of children with language impairment

79% of the participants reported the need for assessing and providing intervention services to clients based on their language background. The multilingual student clinicians who participated in the current study had varying proficiency levels in their non-native languages (as indicated in Table 2). Often, these student clinicians provided services to clients who spoke a language in which the clinician had minimal-moderate proficiency. This is common among students who pursue their undergraduate and graduate education in a state/district different from their native (out-of-state students). The institutes offering speech-language pathology programs are clustered across different states/districts in South India. There are sixteen speech pathology programs in Karnataka (Mangalore - 7, Bangalore - 3, Mysore - 2, Udupi - 1, Dharwad - 1, Kolar - 1, Bellary – 1), seven in Kerala (Kasargod - 2, Kozhikode - 2 centers, Trivandrum – 1, Ernakulum - 1, Palaghat – 1), nine in Tamil Nadu (Chennai - 4, Trichirapalli - 1, Kancheepuram - 2, Kanyakumari – 1, Vellore – 1), and four in Telangana (Secunderabad - 3, Hyderabad - 1 center). Considering the diversity of languages/dialects spoken across South India, it becomes difficult for students from different language backgrounds to adapt to the cultural and linguistic needs of the patients, requiring them to switch between languages while providing clinical service^(4)^.

Narayan^(11)^ reports that although clinicians may have complete subject knowledge about the patient's condition, they cannot adequately convey relevant information to patients from different linguistic backgrounds, especially without a translator. Consequently, clinicians and patients experience communication breakdowns manifesting poor information delivery to the child/child's parent or vice-versa^(12)^. Around 10% of the clinicians in the current study reported using multiple languages while assessing children from multilingual backgrounds. They tend to informally assess the child in the languages spoken at home and school to get a holistic view. Thordardottir^(13)^ recommends the inclusion of all the languages the child may know during an assessment and intervention program to help the child draw all of their available resources rather than restricting them to a subset of their resources. Further, by estimating the proficiency levels of the child in reading, writing, speaking, and understanding each of the languages^(13)^, intervention can be planned by prioritizing a specific language for therapy or by providing an equal emphasis to all the languages known by the child^(3)^.

A considerable number of participants in the study were pursuing their education in the city of Mangalore and encountered speakers of various scripted languages, including Kannada, Malayalam, Tamil, Hindi, and English, as well as unscripted languages like Tulu, Konkani, Beary, and Koraga. At the same time, the Tulu and Konkani had around four (Common Tulu, Brahmin Tulu, Jain dialect, Adivasi dialect) and five dialects (Canara Saraswat, Mangalorean, Nawayathi, Siddi, Kerala Konkani), respectively. Most participants preferred to use the common language used by the parents for assessment and intervention (53.1% and 39.1%, respectively). Around 14.8% of the participants used the language insisted by the parents for assessment and 20.3% for intervention. A small number of participants (12.5%/15.6%) used other languages in the community for assessment and intervention. The assessment and treatment require building a rapport with the child^(14)^. Clinicians often used the parents' preferred language to make the parents actively involved in the sessions^(15)^.

The participants mentioned that they were required to provide therapy in a language different than the native language of the child due to academic reasons, the common language spoken in the community, or the preference of the parent or family member. Students who pursue their education in a non-native state tend to assess/intervene in the language they are familiar with, as the clinician may speak a different native language than the client. Few participants (6.2%/7%) reported using the language spoken in the child's school, the language the clinicians (1.6%/3.9%) were familiar with, and the native language of the child's mother (0.8%/1.6%) for assessment/intervention respectively^(11)^. Research shows that children learning a language other than their home language show delays in areas similar to that of a child with a developmental language disorder^(16)^. These delays often persist until the end of the second year of learning the new language; however, it depends on the language being learned^(16)^. Bilingual children have been diagnosed with a language disorder when they have a specific L2 impairment with scores below the norms in their L1 assessment or poor performance in both languages^(17)^. With no variations introduced in the assessment process while handling multilingual children, over-referrals (patients from bilingual backgrounds being over-identified to have a language disorder due to inadequate developmental expectations) or under-referrals (patients delay in identifying language difficulties when children learn the second language) may be anticipated.

### Factors/Issues surrounding the assessment and intervention of multilingual children with language impairment

Although a certain proportion of student clinicians reported parents' education to not affect assessment and intervention, a majority (65.6%) reported otherwise. Considering the average literacy rate of India is 77.70% (males: 84.70% and females: 70.30%)^(18)^, it becomes less likely to have parents with a strong educational background. Tools deemed for monolingual children become unsuitable for multilingual children due to the norms developed for assessing monolingual children as different from multilingual children^(19)^. Seventy-four (58%) clinicians reported that the currently available multilingual tools seemed insufficient for language assessment, with a major difference in the assessment process for monolingual vs. multilingual children. Clinicians also use informal assessment procedures or available tools, irrespective of the cultural background for which it was developed, to attain a holistic perspective of the individual^(20)^. Likewise, clinicians need to account for the proficiency levels in the available languages, depending on their exposure levels^(21)^. Thordardottir further proposes lowering the cut-off criteria while performing standardized assessments in different languages^(21)^.

The participants in the current study reported that the child/child's parents misunderstood the instructions around 50% of the time leading to communication breakdown during assessment and intervention. The client and the clinician not sharing a common language for communication, misunderstanding the instructions by the interpreter, and lack of experience among interpreters accounted for these communication breakdowns^(22,23)^. Most of the participants were from South India, where multiple languages and dialectal variations are prevalent in the community; hence the student clinicians would have encountered clients who spoke a language or dialect different from theirs, resulting in a communication breakdown. The study results suggest the need for effective translation/interpretation services during the assessment and intervention to address these communication breakdowns. Huang et al.^(24)^ conducted a systematic review to understand how clinicians and interpreters worked together to serve clients from culturally and linguistically different backgrounds. The results indicated that the interpreters were engaged frequently for face- to face assessment sessions compared to intervention. A few challenges reported were the mismatch in the expectations of both the professionals (interpreter & SLPs), the accuracy of the interpretations, the availability of experienced interpreters, and the turnaround time from interpreters.

Additionally, the participants reported using co-clinicians, family members, or sometimes even both as translators during the assessment and/or intervention process. Student co-clinicians who can speak the native language of the client often serve as interpreters. The student co-clinicians understand the instructions and questions related to assessment and treatment better than a family member or interpreter outside the profession. A recent survey of the SLPs in California explored the training and collaborations between SLPs and interpreters^(25)^. Most participants reported using family members/ friends as interpreters, and sometimes minors. However, the participants were satisfied with interpreters' services as most were trained through their employment or previous experiences. There is a scarcity of professional interpreters, and it is crucial to train non-professional interpreters to serve multilingual clients. When professional interpreters are available, their flexibility, turnaround time, and cost are not affordable to the SLPs^(26)^.

### Preparedness of graduate and undergraduate student clinicians in assessing and treating multilingual children with language impairment

Seventy-eight percent (100) of the participants reported that 50-100% of the time, the patients spoke a language in which they were proficient. Students who speak a language similar to that of the client have an advantage over out-of-state students in catering to the clinical needs of the local community. Patients/caregivers build a good rapport with clinicians, providing necessary information regarding the clinical profile of their child and enabling the clinicians to arrive at an appropriate diagnosis. Furthermore, these patients/caregivers tend to understand the clinician's instructions and suggestions and carry out these activities at home^(27)^. On the other hand, out-of-state students find it difficult to request the necessary information about the patient's condition and alleviate their concerns, primarily due to communication breakdowns and language incompetency.

More than half of the participants (55%) felt fairly well prepared to deal with multilingual issues, while the rest felt unsure (37%) or poorly prepared (2%). To overcome these multilingual issues, certain academic programs have incorporated courses dedicated to addressing multicultural and multilingual issues, adopting an informal assessment protocol for clients from the multilingual background and having a student interpreter who is proficient in the client's native (with professional knowledge) on the assessment and treatment team. Stockman et al.^(2)^ reported that students who have taken dedicated courses either become exceptionally or adequately prepared to deal with multilingual issues.

Sixty-four percent of the participants felt that providing clinical services to multilingual populations did eventually help them learn and understand these multilingual issues and handle them appropriately. These reports provide evidence of experience-driven learning. Due to a lack of knowledge of the native language of the client or the absence of the interpreter, the students, throughout their undergraduate and graduate program, attempt to learn the regional language to resolve these clinical challenges. Few academic programs reported that they provide a crash course on regional languages to assist their students in learning the regional language and improving their clinical experience. The clinical mentor/ supervisor, deemed responsible for the students throughout their academic coursework, often offers suggestions and assistance in handling these multilingual issues^(28)^. Past studies reveal that students receiving mentorship perform better than those who didn't receive any^(29)^. A considerable number of clinicians (45%) felt the role of their mentor/colleague impacted their clinical competency over the years drastically. Attending continuing education programs is an ASHA mandate for instructors and clinicians to increase their knowledge about handling various multilingual issues (ASHA, 2018)^(30)^.

### Perceptions of multilingual issues between undergraduate and graduate students

There was a considerable difference in the perceptions and attitudes of graduate students on assessment/intervention compared to undergraduates. These differences were noted on parameters like the significance of multilingual issues, the preparedness, and their perception of these multilingual issues. Many undergraduates reported that the parent's education neither affects the child's assessment nor intervention. With the amount of clinical experience the graduate students (> 5 years) received surpassing the undergraduate students (>3 years), the former group is at an advantage of having deeper analytical skills^(31)^ in clinical management than the latter group. The graduate students nearing the completion of their program tend to be responsible and independent clinicians compared to undergraduates. Graduate students receive additional academic and clinical training and have opportunities to research clinical and related issues. As the participants reported addressing these multilingual issues is experience-driven learning, and graduate students are better prepared to handle similar clinical scenarios efficiently. Graduate students also have more opportunities to attend continuing education programs, in services, and interact with colleagues and peers from other institutes. They understand the need to be a keen learner and keep updating their skill set to serve multilingual clients.

## LIMITATIONS AND FUTURE STUDIES

The current study is one of the first studies that explored the attitudes and perceptions of students in handling multilingual clients. Most of the participants in the study hailed from South India, although the authors attempted to include participants from other parts of the country. Hence the results of the study need to be cautiously generalized. Around 93% of the participants reported being females, similar to the trends in the profession. As this study explores multilingual issues faced by SLPs, particularly handling individuals with language impairments, the results of the study may not be relevant for speech and swallowing-related impairments. It would be interesting to estimate the level of multilingual issues commonly seen in other communication impairments.

The findings of this study assist the academic programs to either integrate concepts on multilingualism and multiculturalism into the curriculum or have in-services to address the same. Additionally, the programs can begin conversations on handling communication breakdowns and support out-of-state clinicians to enhance their clinical experiences. Future studies may explore similar multilingual issues in practicing professionals and faculty. Exploring such practices may be extended to professionals handling other communication disorders (speech sound disorders, literacy, and swallowing disorders).

## CONCLUSION

The current study aimed to explore the perception of Indian student clinicians (undergraduates and graduates) of speech-language pathology in handling multilingual issues. The results conclude that student clinicians often encounter communication breakdowns with patients from diverse cultural and linguistic backgrounds resorting to problems in conducting assessments and interventions and often relying on inexperienced interpreters/translators. Clinicians also reported the unavailability of indigenous multilingual assessment tools, often resorting to western standardized tools. There was a significant difference in the perception levels of undergraduate and graduate students on these multilingual issues.

## Appendix 1 Questionnaire to explore the multilingual issues faced by undergraduate and graduate students in speech-language pathology

**Table:**

Item no.	Questions
**Q1.**	**What are the regional languages spoken in the district where you are studying?**
**Q2.**	**Which of those regional languages have an official script?**
**Q3.**	**How many languages can you understand?** i. >4 (specify), ii. 4 (specify), iii. 3 (specify), iv. 2 (specify), v. 1 (specify)
	**Rate your level of proficiency in each of the languages on a scale from 0 to 10.** (0- none,1-very low, 2-low, 3-fair, 4- slightly less than adequate,5- adequate, 6- slightly more than adequate, 7-good, 8-very good, 9-excellent, 10-perfect)]. (Applies to Q4, Q5, and Q6)
**Q4.**	**How many languages are you proficient in speaking?** i. >4 (specify), ii. 4 (specify), iii. 3 (specify), iv. 2 (specify), v. 1 (specify)
**Q5.**	**How many languages are you proficient in reading?** i. >4 (specify), ii. 4 (specify), iii. 3 (specify), iv. 2 (specify), v. 1 (specify)
**Q6.**	**How many languages are you proficient in writing?** i. >4 (specify), ii. 4 (specify), iii. 3 (specify), iv. 2 (specify), v. 1 (specify)
**Q7.**	**How often do patients who come to your institution/clinic/department for consultation speak the languages you are proficient in?** (i. 0 – 24%, ii. 25 – 49%, iii. 50 – 74%, iv. 75 – 100%)
**Q8.**	**Do you feel that the education of the parent of a child with language impairment would affect your_______?** (i. Assessment, ii. Intervention, iii. Both, iv. Neither assessment nor intervention)
**Q9.**	**Do you feel that there are sufficient tools in Indian languages available to assess multilingual children with language impairment?** (i. Yes, ii. No, iii. Maybe)
**Q10.**	**Do you feel the assessment of children with language impairments should vary depending on whether they are mono/bi/multilinguals?** (i. Always, ii. Most of the times, iii. Sometimes, iv. Never, v. Not sure)
**Q11.**	**Do you feel the intervention of children with language impairments should vary depending on whether they are mono/bi/multilinguals?** (i. Always, ii. Most of the times, iii. Sometimes, iv. Never, v. Not sure)
**Q12.**	**Which language do you commonly use when a multilingual child arrives for assessment?** (i. The common language of the child’s parents, ii. The native language of the child’s mother, iii. The native language of the child’s father, iv. The language spoken in the child’s community, v. The language spoken in child’s school, vi. The language the clinician is familiar with, vii. The language insisted by parents, viii. Others (specify)]
**Q13.**	**Which language do you commonly use when a multilingual child arrives for intervention?** (i. The common language of the child’s parents, ii. The native language of the child’s mother, iii. The native language of the child’s father, iv. The language spoken in the child’s community, v. The language spoken in child’s school, vi. The language the clinician is familiar with, vii. The language insisted by parents, viii. Others (specify)]
**Q14.**	**How often do you face a communication breakdown due to multilingual challenges during the assessment of children with language impairments?** (i. 0 – 24%, ii. 25 – 49%, iii. 50 – 74%, iv. 75 – 100%)
**Q15.**	**How often do you face a communication breakdown due to multilingual challenges during the intervention of children with language impairments?** (i. 0 – 24%, ii. 25 – 49%, iii. 50 – 74%, iv. 75 – 100%)
**Q16.**	**How often did the child misunderstand your instructions during a communication breakdown?** (i. 0 – 24%, ii. 25 – 49%, iii. 50 – 74%, iv. 75 – 100%)
**Q17.**	**How often did the child’s parent misunderstand your instructions during a communication breakdown?** (i. 0 – 24%, ii. 25 – 49%, iii. 50 – 74%, iv. 75 – 100%)
**Q18.**	**In what situation did you use a translator when you and the child shared an uncommon language?** (i. Assessment, ii. Intervention, iii. Both, iv. Neither assessment nor intervention)
**Q19.**	**Who among the following people did you most utilize as a translator for either an assessment/ intervention/ both?** (i. Co-clinician, ii. Family member, iii. Other (specify)]
**Q20.**	**How often did you experience a communication breakdown even though a translator was available?** (i. 0 – 24%, ii. 25 – 49%, iii. 50 – 74%, iv. 75 – 100%)
**Q21.**	**Have you ever experienced a situation where you and the child didn’t share a common language, and a translator was not available?** (i. Yes, ii. No, iii. Sometimes)
**Q22.**	**What is your level of preparation to apply your knowledge to multilingual clients from diverse linguistic backgrounds after having completed the subject/ course?** (i. Exceptionally well prepared, ii. Fairly well prepared, iii. Poorly prepared, iv. Unsure)
**Q23.**	**What experiences have been the most useful to you in learning about multilingual issues?** [a. Taking continuing education seminars/ workshops on multilingual issues, b. Following a mentor/colleague’s recommendation, c. Providing clinical services to multilingual populations, d. Conducting research on the subject of multilingual issues, e. Others (specify)]
